# Attitudes of acceptability and lack of condemnation toward suicide may be predictive of post-discharge suicide attempts

**DOI:** 10.1186/s12888-015-0462-5

**Published:** 2015-04-16

**Authors:** Igor Galynker, Zimri S Yaseen, Jessica Briggs, Fumitaka Hayashi

**Affiliations:** Department of Psychiatry and Behavioral Sciences, Mount Sinai Beth Israel Medical Center, 1st Avenue @ 16th Street, 9-Fierman, New York, NY 10003 USA

**Keywords:** Suicide/Self harm, Anxiety/Anxiety disorders, Cognition, Coping, Ethnicity/race, Impulsivity/Impulse control disorders, Life events/stress

## Abstract

**Background:**

Suicide attempts (SA) after psychiatric hospitalization continue to be a major cause of morbidity. Implicit measures may enhance our ability to assess suicide risk. In this context, we describe the first use of the Suicide Opinion Questionnaire (SOQ) to identify post-discharge suicide attempters.

**Methods:**

Adult psychiatric inpatients admitted for suicidality (N = 91) were administered a battery of measures including the SOQ, and forty were reached and reassessed for SA at two months post-discharge. Exploratory factor analysis (EFA) on items associated with suicidality was performed to identify latent constructs. Linear discriminant analysis (LDA) was used to optimize factor combination for suicide identification. Results were compared with explicit measures of suicidality, and logistic regression was used to control for other risk factors. Finally, a simplified 9-item scale was derived from the results and its performance compared to that of the linear discriminant function.

**Results:**

Twenty items differed between patients with and without SA at intake or follow-up. EFA on these identified two factors: suicide attempters indicated greater acceptability and less moral condemnation of suicide. The LDA-derived discriminant function and 9-item scale was significantly sensitive and specific for post-discharge SA.

**Conclusions:**

Attitudes of acceptability and lack of condemnation toward suicide may constitute an implicit measure of suicidality that could contribute to risk assessment in a high-risk population.

## Background

Suicide is the tenth leading cause of death in the United States, with a rate of 11.3 suicide deaths per 100,000 people [1], and therefore constitutes a significant public health problem. As previously studied, the months following discharge from psychiatric hospitalization represent a period of distinctly elevated suicide risk [2-5]. While lifetime history of suicidal ideation (SI) and suicide attempt (SA) have proven to be the best predictors of suicidality, they remain inadequate [6,7]. Most suicide victims suffer from pre-existing mental health conditions [8,9] and had seen a mental health professional within the six months prior to their suicide [10], yet at present, no combination of factors has demonstrated clinical value in predicting imminent suicide [11]. Thus, one of the most difficult determinations clinicians face is whether a chronically suicidal patient is at acute risk for suicide attempt.

At the heart of the difficulty in predicting future suicides, including near-term suicides, lies the fact that most of the tools used for prospective suicide prediction are based on retrospective, correlational research (reviewed in [7]). Experts in the field have developed twenty or so of these tools, and several were useful in predicting long-term suicidal behavior over a period of 5–20 years in outpatients (Beck Hopelessness Scale [12,13]; Beck Depression Inventory[14,15], Scale for Suicidal Ideation [13], Suicide Intent Scale[16], SNAP-SH subscale [17]) and emergency room patients (SAS [18]). However, these same tools, as well as others, were ineffective in predicting near or long-term SA and completed suicides in high-risk inpatients post-discharge [13,14,19-21]. Only one recent study of the MINI suicidal subscale [22] showed promise in predicting suicide attempts in psychiatric inpatients in the first year post-discharge.

One of the reasons for poor predictive validity of the common scales in psychiatric inpatients is that patients are acutely aware that they are being assessed for their suicidal risk. Those intending to take their own lives often conceal or suppress their suicide intent [23,24]. Promising post-discharge suicide assessment tools do not rely on patients’ self-report of their suicide intent. Instead, they assess either the intensity of the acute suicidal state [25-27] or the psychological aspects leading to this state [28-31]. The latter includes “positive future expectancies”, [28] “self-regulation of unattainable goals”, [29,32], and entrapment [31] or “intrapersonal positive future thinking” [33].

At present no well-validated tool exists for near-term suicide attempt prediction in high-risk inpatients. Ideally, this predictor should not wholly rely on patients’ self-report of SI or intent, as they are often concealed and/or suppressed [23,34], and should employ implicit measures of patients’ cognitive function and attitudes. The implicit association of self with death/suicide was shown to be associated with a six-fold increase in odds of SA in the six months following testing using both the “Suicidal Stroop Test” [35] and the Implicit Association Task (IAT) [36]. While these findings are extremely encouraging, these neurocognitive tasks are technical and time-consuming. Given that implicit association of self with suicide should correspond to greater acceptability of suicide as a legitimate behavioral option, general suicide opinions may add predictive power to suicide risk assessment. The Suicide Opinion Questionnaire (SOQ) probes generalized judgments and beliefs about suicide and thus may be swayed implicitly by respondents’ latent suicidality. Consequently, in our preliminary work we examined if the SOQ could discriminate between attempters and non-attempters in a high risk population.

The Suicide Opinion Questionnaire (SOQ) was originally devised to assess attitudes towards suicide in different cultures and socio-demographic groups. It has been administered to multi-national college students [37-39], medical students [40], and mental health professionals [41]. However, it has been assessed in relation to suicidality in only one previous study [42]. In that study, the SOQ was administered to 738 undergraduate volunteers, to compare individuals with and without a lifetime history of suicide attempt(s) or suicidal ideation. SOQ responses differed between groups on items primarily having to do with acceptability of suicide, as well as religion, intent, degree of control, anger, and societal influence. In addition, the responses were used to generate a discriminant function that was able to differentiate a respondent’s suicide history using only the SOQ responses with some level of accuracy.

In light of the above it appears that the SOQ, although it does not contain questions explicitly probing lifetime history of SA and SI, may do so implicitly by probing patients’ permissive vs. prohibitive attitudes towards suicide as a legitimate solution for life’s problems. As such, the SOQ or its subscales have a potential to be useful in prospectively identifying those at risk for suicide, whether alone or as a module in a multimodal assessment of suicide risk [43]. As a suicide risk assessment tool, the SOQ would be most useful when used with high-risk patients, such as those admitted to an acute psychiatric unit for SA or dangerousness to self.

In this context, we set out to test whether the SOQ, its subscales, or its individual items would discriminate future suicide attempters among high-risk patients prior to their discharge from an inpatient psychiatric unit. We hypothesized that we would be able to derive a subscale of the SOQ that would associate with post-discharge SA, and that such a subscale would rely primarily on items relating to the acceptability of suicide. We tested this hypothesis by administering the SOQ to patients admitted to the hospital for suicidal attempt or ideation and re-evaluating the patients for suicide attempt(s) within two months of discharge.

## Methods

### Study subjects

This study was performed as part of a larger study at our institution in which patients hospitalized for suicidality were assessed prospectively for factors that may help in the prediction of a suicide attempt within two months (the STS Study) [44]. The STS Study was approved by the Beth Israel Medical Center Institutional Review Board and is in compliance with the code of ethics of the World Medical Association. Informed consent was obtained from all subjects after the nature of the procedures was explained. Subjects in the study satisfied four inclusion criteria: 1) age was limited to between 18 and 65 years old, as prior studies suggest there are differences in suicidal behavior in adolescents and the elderly compared to the general population [45], 2) subjects must have been admitted for dangerousness to self either due to suicidal ideation or suicide attempt, 3) subjects must have been able to understand the nature and substance of the informed consent to participate in this study, 4) subjects needed to have at least two verifiable collateral contacts to improve tracking for subsequent assessment.

Additionally, in order to be included in the study, there were two exclusion criteria which patients could not satisfy. Patients with mental retardation, cognitive impairment, or linguistic limitations which precluded their understanding of the informed consent or research questions were excluded, as were patients with significant medical or neurological disorders and possible delirium.

Suicidal ideation was defined as a positive answer to the question “Have you ever seriously thought about committing suicide?”, as in the National Comorbidity Study [46]. Suicide attempt was defined according to the Columbia Suicide Severity Rating Scale (C-SSRS) as “A potentially self-injurious act committed with at least some wish to die, *as a result of act;* behavior was in part thought of as method to kill oneself”. This definition was used both when applying the inclusion criteria and at follow-up [47].

Psychiatrist-determined clinical diagnoses for study participants were gathered from patient discharge summaries. Psychiatrists were experienced, board-certified staff psychiatrists or psychiatry residents directly supervised by the former. Following our previously used methodology, diagnoses were condensed into four categories to maximize degrees of freedom, (thereby increasing statistical power in subsequent analyses) as well as diagnostic reliability [48-50]. DSM-IV Axis I diagnoses were coded as 1) No primary DSM-IV Axis I mood, anxiety, or psychotic disorder (this category comprised primary diagnoses of Borderline personality d/o, adjustment and substance induced disorders, primary diagnoses of substance dependence, and diagnoses recorded as “Mood disorder not otherwise specified”), 2) Anxiety or unipolar depressive disorders, 3) Bipolar I, II, or NOS disorders, and 4) Psychotic disorders.

### Questionnaires

We describe the results of three questionnaires administered to our study subjects: SOQ [51,52], Beck Scale for Suicide Ideation (BSS) [20,53], and Columbia Suicide Severity Rating Scale (C-SSRS) [47]. The SOQ (Suicide Opinion Questionnaire) is a self-administered, 100-item questionnaire developed by Domino et al. In our study, we used the 1982 version [52]. Each item consists of a statement regarding an attitude towards suicide, for which the patient is asked to select one of five possible answers: A - Strongly Agree, B- Agree, C-Undecided, D- Disagree, or E - Strongly Disagree. For the purposes of data manipulation, these letters were changed to numbers 1 through 5 during data analysis. Though the factor-structure of the SOQ has not proven to be stable between populations, thus warranting an exploratory factor analytic approach in a novel population, test-retest reliability has been high [54]. The BSS [20,53] is a self-administered scale with 21 items that measures suicidal ideation, and the C-SSRS [47] is a measure of the severity of suicidal ideation and behavior, administered by a trained rater.

Responses from 91 patients were analyzed. Due to the fact that the SOQ was introduced into an ongoing study after 25 subjects were already recruited, the first 25 patients were given the SOQ at the time of follow-up (two months after discharge), while the remaining 66 patients were administered the questionnaire at study intake. Because the SOQ has been shown to assess a trait measure, and is thus stable over time [51], we have combined the data from both groups to increase power. Suicidal behavior was assessed at admission, over lifetime, preceding SOQ administration (at admission for SOQ administered during inpatient hospitalization (n = 66) and post-discharge for SOQ administered at follow-up (n = 25)), and post-discharge (assessed prospectively at admission (n = 66) or retrospectively at follow-up (n = 25)).

### Follow-up

Patients were contacted via phone two months after discharge from the hospital; 40 agreed to follow-up interview while 51 refused or were lost to follow-up. Those who had agreed to continue participation in the study, were asked to come in to the research office to be assessed for suicide attempts following discharge using the C-SSRS, which was administered by trained research assistants.

### Statistical analysis

All statistical analyses were performed using SPSS. In order to identify the SOQ subscale best able to identify acute suicidality, we identified all items differing at a p < 0.05 level between those with SA vs. No SA, at time of admission and post-discharge, using univariate ANOVA F-tests. Maximum Likelihood Factor analysis (with Varimax factor rotation) of all items thus identified as differing at the p < 0.05 level was performed to identify latent dimensions. Scores on each factor were then entered in a linear discriminant analysis. ROC analysis was used to examine the diagnostic power of the resultant discriminant function score to identify Recent and Post-discharge suicide attempters. Finally binary logistic regression was used to control for age, gender, substance abuse, C-SSRS-measured severity of SI at admission, and diagnostic category. (In addition, a simplified version of the discriminant function, amenable to easy pencil and paper calculation, was devised based on item loadings on each factor; the diagnostic power of the resulting 9-item scale was compared to the discriminant function).

To determine to what extent the SOQ assessed suicidality independent of overt suicidal ideation, pairwise correlations between the SOQ, C-SSRS-measured severity of SI, and BSS total scores were calculated (using Pearson correlation for continuous variable pairs and Spearman rank correlation for C-SSRS-pairs).

Finally, as a comparison to the SOQ, the BSS and C-SSRS were examined as possible predictors of post-discharge SA.

## Results

### Demographics

91 subjects were assessed, and 40 were followed-up at two months post discharge. Of those followed up, 7 reported a suicide attempt in the 2-month follow up interval (5 by overdose/poisoning, 1 by overdose/poisoning and hanging, and 1 by asphyxiation). Table 1 shows the demographic characteristics of our study population and comparison of those reachable for follow-up and those lost to follow-up on core demographic and clinical measures. The average age of the participants (39.1 years) appears similar to previously published studies involving patients with histories of suicidality [55]. The demographic variables are similar to those found in other studies utilizing our clinical population [44]. Those reached for follow up did not differ significantly from those not reachable for follow-up, except in the distribution of diagnoses (Table 1); those lost to follow-up were significantly more likely to have no primary psychotic or major mood or anxiety-disorder diagnosis.

**Table 1:** **Demographics**

		Total		Reachable for follow-up?			
				No		Yes	
Initial SA		N	%	N	%	N	%
	No attempt at admit	52	57.1%	29	59.2%	23	54.8%
	Attempt leading to admit	39	42.9%	20	40.8%	19	45.2%
Sex		N	%	N	%	N	%
	Female	50	55.6%	30	62.5%	20	47.6%
	Male	40	44.4%	18	37.5%	22	52.4%
Race		N	%	N	%	N	%
	American Indian	1	1.1%	1	2.1%	0	0%
	Asian	7	7.9%	4	8.3%	3	7.3%
	Black	18	20.2%	12	25.0%	6	14.6%
	Pacific Islander	2	2.2%	2	4.2%	0	0%
	White	44	49.4%	24	50.0%	20	48.8%
	Other	17	19.1%	5	10.4%	12	29.3%
Ethnicity		N	%	N	%	N	%
	Hispanic	22	24.7%	8	66.7%	14	34.1%
	Non-Hispanic	67	75.3%	4	33.3%	27	65.9%
Marital status		N	%	N	%	N	%
	Single	80	90.9%	43	87.8%	37	94.9%
	Married	8	9.1%	6	12.2%	2	5.1%
Housing		N	%	N	%	N	%
	Undomiciled	8	9.1%	3	6.1%	5	12.8%
	Domiciled	80	90.9%	46	93.9%	34	87.2%
History of incarceration		N	%	N	%	N	%
	Never incarcerated	60	66.7%	30	61.2%	30	73.2%
	Ever incarcerated	30	33.3%	19	38.8%	11	26.8%
Primary diagnosis		N	%	N	%	N	%
	Psychotic Disorder	10	11%	5	10.2%	5	11.9%
	Bipolar (non-psychotic)	15	16.6%	11	22.4%	4	9.5%
	Unipolar and Anxiety	44	48.4%	18	36.7%	26	61.9%
	No primary mood, anxiety, or psychotic disorder	22	24.2%	15	30.6%	7	16.7%
Substance abuse		N	%	N	%	N	%
	No substance abuse	35	38.5%	16	32.7%	19	45.2%
	Any substance abuse	56	61.5%	33	67.3%	23	54.8%
Scalar demographics		Mean	SD*	Mean	SD	Mean	SD
	Age	37.8	13.2	36.6	13.5	39.1	12.9
	Years of education	13.1	2.7	13.5	2.4	12.7	3.0

*SD = Standard Deviation.

### Item identification

Twenty items that differed at a p < 0.05 level were identified in univariate ANOVA F-tests. Four items were associated only with SA at admission, fourteen with SA post-discharge, and two were associated with both conditions (see Table 2).

**Table 2:** **SA-associated SOQ item identification**

SOQ item number	a) SA at admission	b) SA post-discharge
24		x
26		x
39		x
44		x
45	x	
51		x
52		x
57	x	
59		x
67	x	x
75	x	x
78	x	
79		x
80		x
86		x
87		x
89		x
92		x
95	x	
99		x

‘x’ designates item mean differences between SA and no SA groups significant at the p < 0.05 level in univariate ANOVA.

### Factor analysis of SOQ items differentially associated with suicidality

**Figure 1:**
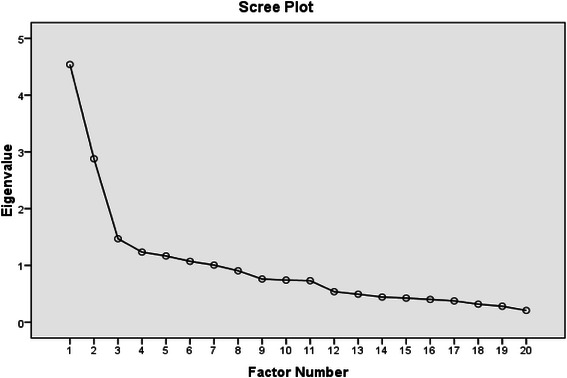
Scree-plot for exploratory factor analysis showing eigenvalues (y-axis) for derived factors (x-axis).

In our exploratory maximum likelihood factor analysis of the above items, we found Bartlett’s test of sphericity was significant (p < 0.0005) and the Keyser-Meyer-Olkin measure of sampling adequacy was adequate at 0.734. Examination of the scree-plot indicated a 2 factor solution (see Figure 1).

This solution accounted for 30% of the variance and had adequate fit to the data (Chi-squared = 175.03, DF = 151, p = 0.088).

Items loading strongly on the first factor (loading > 0.5) primarily address the personal acceptability of suicide, while those loading strongly on the second (loading > 0.5) primarily address religious/moral disapproval of suicide. Factor loadings of items are shown in Table 3, below. Mean scores on factor one were lower (indicating greater acceptability) for subjects with (vs. without) SA at admission, SA post-discharge, SA preceding SOQ administration, and lifetime SA. Mean scores on factor two were higher (indicating less disapproval) for subjects with (vs. without) SA at admission, SA preceding SOQ administration, and lifetime SA.

**Table 3:** **Rotated factor matrix** ^**a**^

SOQ item #	Item	Factor	
		1	2
67	Sometimes suicide is the only escape from life’s problems.	**.601**	-.248
87	People who die by suicide should not be buried in the same cemetery as those who die naturally.	**.601**	.351
79	We should have “suicide clinics” where people who want to die could do so in a painless and private manner	**.584**	-.318
92	Some people are better off dead.	**.582**	-.364
86	Suicide occurs only in civilized societies.	**.569**	.244
59	Suicide is normal behavior.	**.501**	-.049
52	Improvement following a suicidal crisis indicates that the risk is over.	.493	.253
99	Suicide is much more frequent in our world today than it was in early cultures such as Egypt, Greece, and the Roman Empire.	.475	.217
44	The possibility of committing suicide is greater for older people (those 60 and over) than for younger people (20 to 30).	.467	-.172
89	Children from larger families (i.e., three or more children) are less likely to commit suicide as adults than single or only children.	.460	.067
24	John Doe, age 45, has just committed suicide. An investigation will probably reveal that he has considered suicide for quite a few years.	.447	.039
26	The suicide rate among physicians is substantially greater than for other occupational groups.	.437	.074
80	Those people who attempt suicide are usually trying to get sympathy from others.	.407	.270
51	The suicide rate is higher for minority groups such as Chicano, American Indian, and Puerto Ricans than for Whites.	.394	.156
45	Most people who commit suicide do not believe in an afterlife.	.316	.103
39	The method used in a given suicide probably reflects whether the action was impulsive or carefully and rationally planned.	.295	-.073
78	Suicide goes against the laws of God and/or of nature.	-.028	**.778**
57	In general, suicide is an evil act not to be condoned.	.115	**.677**
95	People do not have the right to take their own lives.	.002	**.584**
75	Usually, relatives of a suicide victim had no idea of what was about to happen.	.048	.468

Extraction Method: Maximum Likelihood. Rotation Method: Varimax with Kaiser Normalization. ^a^Rotation converged in 3 iterations. Item loadings above 0.5 are in bold.

### Discriminant analysis of SOQ results

**Figure 2:**
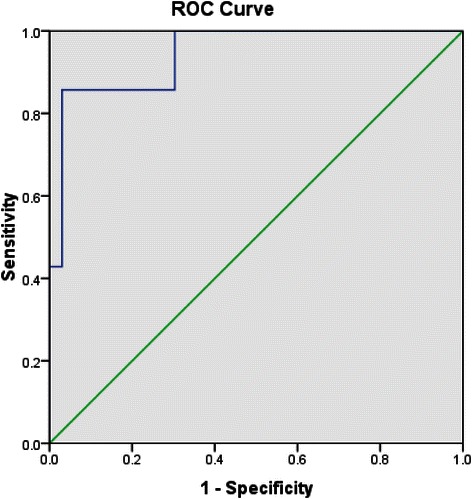
Receiver-operator characteristic function for LDA-derived discriminant function for identification of post-discharge SA. The curve illustrates the sensitivity (y-axis) and 1-specificity (x-axis) for identification of post-discharge SA for successive cut-scores on the discriminant function.

Factor scores derived from the above analysis were then entered into a linear discriminant analysis to determine optimal weighting for discrimination of post-discharge suicide attempters from non-attempters. ROC analysis of the discriminant function score was used to examine the discriminant function behavior and diagnostic power. The canonical discriminant function coefficients were 1.0 for factor 1 and −0.15 for factor 2. Cross validated correct classification rate on 7 attempters and 33 non-attempters was 87.5% (35/40 subjects correctly classified). AUC for the ROC was 0.944, asymptotic p < 0.0005 (see Figure 2). At the optimal cut-score, sensitivity was 85.7% and specificity 97%. For SA preceding SOQ administration, the ROC analysis remained significant with AUC = 0.684, p = 0.005 on 30 attempters and 56 non-attempters). Here sensitivity and specificity at the optimal cut-score were 70% and 62.5%, respectively, with 56/86 subjects classified correctly.

### Logistic regression of the discriminant function

The discriminant function remained a statistically significant predictor in binary logistic regression with post-discharge SA as the outcome variable after control for age, sex, substance abuse, severity of SI as measured by C-SSRS at admission, and diagnostic category. These results are summarized in Table 4. (Note that because lower scores on the discriminant function are associated with post-discharge SA, beta values for the discriminant function are negative).

**Table 4:** **Logistic regression for post-discharge SA**

	Variables in the equation					
	B	S.E.	Wald	df	Sig.	Exp(B)
Diagnosis*			.017	3	.999	
Psychotic	-.497	3.932	.016	1	.899	.608
Bipolar	−14.600	25101.987	.000	1	1.000	.000
Unipolar/Anxiety	-.096	2.065	.002	1	.963	.908
Age	.051	.107	.232	1	.630	1.053
Male gender**	4.845	3.147	2.370	1	.124	127.059
Substance use***	-.211	2.191	.009	1	.923	.810
SI severity	-.543	.936	.336	1	.562	.581
**Discriminant function**	**−5.510**	**2.603**	**4.479**	**1**	**.034**	**.004**
Constant	−8.101	8.396	.931	1	.335	.000

*Reference category: No Axis I Major Mood or Psychotic D/O. **Reference category: Female gender. ***Reference category: Alcohol or Drug abuse present.

### Relation of the discriminant function to simplified 9-item score

A simplified 9-item score was calculated as the sum of scores for items loading above 0.5 on factor one minus the sum of scores for items loading above 0.5 on factor two. Pearson correlation between this simplified 9-item score and the discriminant function was expectably very strong (r = 0.80, two tailed p < 0.0005). AUC for the ROC was somewhat lower at 0.861, asymptotic p = 0.003. At the optimal cut-score (<10), sensitivity was the same at 85.7% and specificity reduced to 69.7%. For SA preceding SOQ administration, the ROC analysis remained significant with AUC improved to 0.769, p < 0.0005 on 30 attempters and 57 non-attempters). Here sensitivity and specificity at the optimal cut-score (<10) were improved to 80% and 66.7%, respectively.

### Relation of the SOQ discriminant function to C-SSRS and BSS

The discriminant function did not correlate significantly with C-SSRS ranked severity of SI nor with BSS total score. Spearman rank correlation between BSS and C-SSRS suicidality measures was significant (two tailed p = 0.005) but moderate (rho = 0.337). Factor analysis (data not shown) of the BSS suggested a single factor solution so BSS total score was used as a candidate predictor of post-discharge SA. Contrary to what might be expected, *lower* BSS scores showed a non-significant trend to increased risk of post-discharge SA (AUC = 0.650, p = 0.292). C-SSRS rating of SI severity showed no relation with post discharge SA (AUC = 0.521, p = 0.856).

## Discussion

In this paper we report a novel use of the Suicide Opinion Questionnaire (SOQ) in a clinical population to discriminate post-discharge suicide attempters from non-attempters in a high-risk sample – psychiatric inpatients admitted with suicidal ideation and/or attempt(s). Items from two SOQ-derived factors, relating to acceptability of suicide and to religious/moral condemnation of suicide, appear to be a promising screening tool to determine the likelihood of suicide attempt within two months of discharge. Thus, potentially, a 9-item SOQ subscale could be a measure of implicit suicide risk, which would add another dimension to previously reported methods for evaluation of imminent suicide risk [35,36,44,56]. Analysis of the SOQ answers indicates that the pattern of patients’ responses is sensitive to post-discharge and recent SA and may be useful in the prediction of suicide risk during the first two months following discharge. The ability to correctly classify post-discharge suicidality is largely due to 9 out of the 100 questions that make up the SOQ. Moreover, the 9 items appear to reflect two underlying factors - acceptability of suicide and religious/moral prohibition. Participants who made a suicide attempt within two months of discharge tended to endorse statements describing suicide as acceptable and even normal, while disagreeing with statements that referenced religious and/or moral prohibition of suicide. These results are supportive of our hypothesis that SOQ items assessing generalized suicide might discriminate recent suicide attempters and aid in the prediction of post-discharge SA.

Patients who suffered only from suicidal ideation (and did not make a post-discharge attempt) did not endorse items promoting the acceptability and normalcy of suicide, and agreed with statements objecting to suicide on moral and/or religious grounds. Moreover, explicit assessments of suicidality (BSS, C-SSRS) did not demonstrate significant association with recent or post-discharge SA. This suggests that SOQ-assessed general acceptability of suicide may capture an implicit personal acceptance of SA as a problem-solving or coping strategy in this population. Therefore, it is possible that what distinguishes imminent suicide attempters from the much larger group of patients with suicidal ideation is an implicit acceptance of the concept of suicide. The SOQ might thus be used to measure internal association with suicide, much like the Suicidal Stroop Test and IAT, while being significantly simpler to administer.

Our findings, though novel, are in accordance with the literature, particularly Nock and Banaji’s 2007 finding that self-association with self-injury and suicide differentiated adolescents with recent and imminent suicide attempts from those with suicide ideation alone. The authors pointed out that most people who suffer from depression and hopelessness will not make a suicide attempt, and suggested that this may be because they do not see self-injury as a part of their “behavioral repertoire”. They went on to suggest the possibility that as individuals think more seriously about suicide, they begin to personally associate with the act itself, which makes them more likely to make an actual attempt [57]. Our results agree with this finding in that those who endorse statements promoting the acceptability of suicide are much more likely to make an attempt within two months of discharge. Thus our data suggest that those who self-associate with and accept the idea of suicide are at a significantly higher risk for suicide attempt. However, it should be noted that the degree of correlation between scores on the SOQ subscale we identify here and IAT and Stroop tests of suicidality remains to be determined.

Of note, our results indicate that in a clinical population of psychiatric inpatients with high suicide risk, only two of the factors described in the literature [42] seem to contribute to the scale’s predictive value: acceptability and religion. The reasons why explicit suicide intent, anger, degree of control, and societal influence, which differentiated students with and without past lifetime SA, were not useful in identifying post-discharge suicide attempters within high-risk inpatients remain to be examined in future studies.

Our study has a number of limitations. The first is the small number of patients involved in this preliminary report; the study needs to be replicated with a larger group of high-risk psychiatric inpatients. An additional limitation lies in the generalizability of our results. Our inpatient demographics reflect our local patient population of urban, low-income households. To determine the effects of demographic differences on our results, we would need to replicate our study using different high risk patient populations, including but not limited to more rural and/or higher income groups, and those of different cultures. This latter aspect is of particular importance as SOQ responses in non-clinical populations did show significant differences between different nations and cultures [37-39]. Another limitation is that the SOQ was administered at different time points for some of our patients (at two months post-discharge vs. at admission), which could confound the study if the SOQ is acutely state-sensitive. However, our assumption that the SOQ measures trait opinions is based on test-retest analyses which concluded that it is a relatively stable measure [51]. Still, a test-retest analysis needs to be replicated in a high risk clinical population.

## Conclusion

Within its limitations, this report suggests that the SOQ-derived 9-item subscale assessing acceptability and religious/moral condemnation of suicide appears to be a promising screening tool to determine the likelihood of suicide attempt within two months of discharge for high-risk patients hospitalized for suicidal ideation or attempt. Such an examination would improve clinicians’ ability to distinguish patients at acute risk for an imminent suicide attempt from other high risk patients who may not be at imminent risk. Additional prospective replication is needed to further refine this approach and establish its possible place in clinicians’ evaluations of high risk suicidal patients.

## References

[1] Centers for Disease Control and Prevention (CDC). “Web-based injury statistics query and reporting system (WISQARS)”. National Center for Injury Prevention and Control, Centers for Disease Control and Prevention [2010 Jun 14]. (2010). Available at: www.cdc.gov/injury.

[2] Pirkola S, Sohlman B, Wahlbeck K (2005). The characteristics of suicides within a week of discharge after psychiatric hospitalisation: a nationwide register study. BMC Psychiatry.

[3] Appleby L, Shaw J, Amos T, McDonnell R, Harris C, McCann K (1999). Suicide within 12 months of contact with mental health services: national clinical survey. BMJ.

[4] Goldacre M, Seagroatt V, Hawton K (1993). Suicide after discharge from psychiatric inpatient car. Lancet.

[5] Qin PNM (2005). Suicide risk in relation to psychiatric hospitalization: Evidence based on longitudinal registers. Arch Gen Psychiat.

[6] Cochrane-Brink KA, Lofchy JS, Sakinofsky I (2000). Clinical rating scales in suicide risk assessment. Gen Hosp Psychiatry.

[7] Roos L, Sareen J, Bolton JM (2013). Suicide risk assessment tools, predictive validity findings and utility today: time for a revamp?. Neuropsychiatry.

[8] Nock MK, Deming CA, Fullerton CS, Gilman SE, Goldenberg M, Kessler RC (2013). Suicide among soldiers: a review of psychosocial risk and protective factors. Psychiatry.

[9] Nock MK, Hwang I, Sampson NA, Kessler RC (2010). Mental disorders, comorbidity and suicidal behavior: results from the national comorbidity survey replication. Mol Psychiatry.

[10] Matthews K, Milne S, Ashcroft GW (1994). Role of doctors in the prevention of suicide: the final consultation. Br J Gen Pract.

[11] Large M, Sharma S, Cannon E, Ryan C, Nielssen O (2011). Risk factors for suicide within a year of discharge from psychiatric hospital: a systematic meta-analysis. Aust N Z J Psychiatry.

[12] Beck AT, Brown G, Berchick RJ, Stewart BL, Steer RA (1990). Relationship between hopelessness and ultimate suicide: a replication with psychiatric outpatients. Am J Psychiatry.

[13] Brown G, Beck A, Steer R, Grisham J (2000). Risk factors for suicide in psychiatric outpatients: a 20-year prospective study. J Consult Clin Psych.

[14] Beck A, Weishaar M (1990). Suicide risk assessment and prediction. Crisis.

[15] Beck AT, Steer RA, Ball R, Ranieri W (1996). Comparison of beck depression inventories -IA and -II in psychiatric outpatients. J Pers Assess.

[16] Stefansson J, Nordstrom P, Jokinen J (2012). Suicide intent scale in the prediction of suicide. J Affect Disord.

[17] Yen S, Shea MT, Walsh Z, Edelen MO, Hopwood CJ, Markowitz JC (2011). Self-harm subscale of the schedule for nonadaptive and adaptive personality (SNAP): predicting suicide attempts over 8 years of follow-up. J Clin Psychiatry.

[18] Waern M, Sj√∂str√∂m N, Marlow T, Hetta J (2010). Does the suicide assessment scale predict risk of repetition? a prospective study of suicide attempters at a hospital emergency department. Eur Psychiat.

[19] Beck AT, Steer RA, Kovacs M, Garrison B (1985). Hopelessness and eventual suicide: a 10-year prospective study of patients hospitalized with suicidal ideation. Am J Psychiatry.

[20] Beck AT, Steer RA, Ranieri WF (1988). Scale for suicide ideation: psychometric properties of a self-report version. J Clin Psychol.

[21] Bolton JM, Spiwak R, Sareen J (2012). Predicting suicide attempts with the SAD PERSONS scale: a longitudinal analysis. J Clin Psychiatry.

[22] Roaldset JO, Linaker OM, Bjorkly S (2012). Predictive validity of the MINI suicidal scale for self-harm in acute psychiatry: a prospective study of the first year after discharge. Arch Suicide Res Off J Int Acad Suicide Res.

[23] Horesh N, Zalsman G, Apter A (2004). Suicidal behavior and self-disclosure in adolescent psychiatric inpatients. J Nerv Ment Dis.

[24] Busch KA, Fawcett J, Jacobs DG (2003). Clinical correlates of inpatient suicide. J Clin Psychiatry.

[25] Yaseen Z, Katz C, Johnson M, Eisenberg D, Cohen L, Galynker I (2010). Construct development: the suicide trigger scale (STS-2), a measure of a hypothesized suicide trigger state. BMC Psychiatry.

[26] Yaseen ZS, Gilmer E, Modi J, Cohen LJ, Galynker II (2012). Emergency room validation of the revised suicide trigger scale (STS-3): a measure of a hypothesized suicide trigger state. PLoS One.

[27] Beck AT, Kovacs M, Weissman A (1979). Assessment of suicidal intention: the Scale for Suicide Ideation. J Consult Clin Psychol.

[28] O’Connor RC, Fraser L, Whyte MC, Machale S, Masterton G (2008). A comparison of specific positive future expectancies and global hopelessness as predictors of suicidal ideation in a prospective study of repeat self-harmers. J Affect Disord.

[29] O’Connor RC, Fraser L, Whyte MC, MacHale S, Masterton G (2009). Self-regulation of unattainable goals in suicide attempters: the relationship between goal disengagement, goal reengagement and suicidal ideation. Behav Res Ther.

[30] O’Connor E, Gaynes BN, Burda BU, Soh C, Whitlock EP (2013). Screening for and treatment of suicide risk relevant to primary care: a systematic review for the U.S. Preventive Services Task Force. Ann Intern Med.

[31] O’Connor RC, Smyth R, Ferguson E, Ryan C, Williams JM (2013). Psychological processes and repeat suicidal behavior: a four-year prospective study. J Consult Clin Psych.

[32] O’Connor RC, O’Carroll RE, Ryan C, Smyth R (2012). Self-regulation of unattainable goals in suicide attempters: a two year prospective study. J Affect Disord.

[33] O’Connor RC, Smyth R, Williams JM (2015). Intrapersonal positive future thinking predicts repeat suicide attempts in hospital-treated suicide attempters. J Consult Clin Psychol.

[34] Horesh N, Apter A (2006). Self-disclosure, depression, anxiety, and suicidal behavior in adolescent psychiatric inpatients. Crisis.

[35] Cha CB, Najmi S, Park JM, Finn CT, Nock MK (2010). Attentional bias toward suicide-related stimuli predicts suicidal behavior. J Abnorm Psychol.

[36] Nock MK, Park JM, Finn CT, Deliberto TL, Dour HJ, Banaji MR (2010). Measuring the suicidal mind implicit cognition predicts suicidal behavior. Psychol Sci.

[37] Domino G, Cohen A, Gonzalez R (1981). Jewish and Christian attitudes on suicide. J Relig Health.

[38] Domino G, Leenaars AA (1989). Attitudes toward suicide: a comparison of Canadian and U.S. college students. Suicide Life Threat Behav.

[39] Domino G (2005). Cross-cultural attitudes towards suicide: the SOQ and a personal odyssey. Arch Suicide Res.

[40] Domino G, Takahashi Y (1991). Attitudes toward suicide in Japanese and American medical students. Suicide Life Threat Behav.

[41] Domino G, Swain BJ (1985). Recognition of suicide lethality and attitudes toward suicide in mental health professionals. OMEGA--J Death Dying.

[42] Limbacher M, Domino G (1985). Attitudes toward suicide among attempters, contemplators, and nonattempters. OMEGA--J Death Dying.

[43] Galynker I, Yaseen Z, Briggs J (2014). Assessing risk for imminent suicide. Psychiatr Ann.

[44] Yaseen ZS, Kopeykina I, Gutkovich Z, Bassirnia A, Cohen LJ, Galynker II (2014). Predictive validity of the suicide trigger scale (STS-3) for post-discharge suicide attempt in high-risk psychiatric inpatients. PLoS One.

[45] Brent DA, Baugher M, Bridge J, Chen T, Chiappetta L (1999). Age- and sex-related risk factors for adolescent suicide. J Am Acad Child Psy.

[46] Kessler RC, Borges G, Walters EE (1999). Prevalence of and risk factors for lifetime suicide attempts in the National Comorbidity Survey. Arch Gen Psychiatry.

[47] Posner K, Brown GK, Stanley B, Brent DA, Yershova KV, Oquendo MA (2011). The Columbia–suicide severity rating scale: initial validity and internal consistency findings from three multisite studies with adolescents and adults. Am J Psychiatry.

[48] Lieberman PB, Baker FM (1985). The reliability of psychiatric diagnosis in the emergency room. Hosp Commun Psychiatry.

[49] Warner MD, Peabody CA (1995). Reliability of diagnoses made by psychiatric residents in a general emergency department. Psychiatr Serv.

[50] Cheniaux E, Landeira-Fernandez J, Versiani M (2009). The diagnoses of schizophrenia, schizoaffective disorder, bipolar disorder and unipolar depression: interrater reliability and congruence between DSM-IV and ICD-10. Psychopathology.

[51] Domino G (1996). Test-retest reliability of the suicide opinion questionnaire. Psychol Rep.

[52] Domino G, Moore D, Westlake L, Gibson L (1982). Attitudes toward suicide: a factor analytic approach. J Clin Psychol.

[53] Hersen M, Hilsenroth MJ, Segal DL (2003). Comprehensive Handbook of Psychological Assessment, Personality Assessment, vol. 2.

[54] Kodaka M, Poštuvan V, Inagaki M, Yamada M (2011). A systematic review of scales that measure attitudes toward suicide. Int J Soc Psychiatry.

[55] Ayer DW, Jayathilake K, Meltzer HY (2008). The InterSePT suicide scale for prediction of imminent suicidal behaviors. Psychiatry Res.

[56] Yaseen ZS, Briggs J, Kopeykina I, Orchard KM, Silberlicht J, Bhingradia H (2013). Distinctive emotional responses of clinicians to suicide-attempting patients - a comparative study. BMC Psychiatry.

[57] Nock MK, Banaji MR (2007). Prediction of suicide ideation and attempts among adolescents using a brief performance-based test. J Consult Clin Psychol.

